# Mania and bipolar depression: complementing not opposing poles—a post-hoc analysis of mixed features in manic and hypomanic episodes

**DOI:** 10.1186/s40345-021-00241-5

**Published:** 2021-11-16

**Authors:** Christoph Born, Heinz Grunze, Robert M. Post, Lori L. Altshuler, Ralph Kupka, Susan L. McElroy, Mark A. Frye, Trisha Suppes, Paul E. Keck, Willem A. Nolen, Lars Schaerer

**Affiliations:** 1 Paracelsus Medical University, Nuremberg, Germany; 2 Psychiatrie Schwäbisch Hall, Ringstrasse 1, 74523 Schwäbisch Hall, Germany; 3 Bipolar Collaborative Network, Bethesda, MD USA; 4 Department of Psychiatry and Behavioral Sciences, George Washington University, Washington, DC USA; 5 Department of Psychiatry and Biobehavioral Sciences, David Geffen School of Medicine, University of California, Los Angeles, CA USA; 6 Department of Psychiatry, VA Greater Los Angeles Healthcare System, West Los Angeles Healthcare Center, Los Angeles, CA USA; 7 Department of Psychiatry, Amsterdam UMC, Vrije Universiteit, Amsterdam, The Netherlands; 8 Lindner Center of HOPE, Mason, OH USA; 9 Biological Psychiatry Program, University of Cincinnati Medical College, Cincinnati, OH USA; 10 Department of Psychiatry and Psychology, Mayo Clinic, Rochester, MN USA; 11 Department of Psychiatry and Behavioral Sciences, Stanford University School of Medicine, Palo Alto, CA USA; 12 V.A. Palo Alto Health Care System, Palo Alto, CA USA; 13 Department of Psychiatry, University Medical Center Groningen, University of Groningen, Groningen, The Netherlands; 14 Department of Psychiatry and Psychotherapy Medical Center, Faculty of Medicine, University of Freiburg, Freiburg im Breisgau, Germany

**Keywords:** Bipolar disorder, Depression, Hypomania, Mania, Mixed states

## Abstract

**Background:**

Depending on the classification system used, 5–40% of manic subjects present with concomitant depressive symptoms. This post-hoc analysis evaluates the hypothesis that (hypo)manic subjects have a higher burden of depression than non-(hypo)manic subjects.

**Methods:**

Data from 806 Bipolar I or II participants of the Stanley Foundation Bipolar Network (SFBN) were analyzed, comprising 17,937 visits. A split data approach was used to separate evaluation and verification in independent samples. For verification of our hypotheses, we compared mean IDS-C scores ratings of non-manic, hypomanic and manic patients. Data were stored on an SQL-server and extracted using standard SQL functions. Linear correlation coefficients and pivotal tables were used to characterize patient groups.

**Results:**

Mean age of participants was 40 ± 12 years (range 18–81). 460 patients (57.1%) were female and 624 were diagnosed as having bipolar I disorder (77.4%) and 182 with bipolar II (22.6%). Data of 17,937 visits were available for analyses, split into odd and even patient numbers and stratified into three groups by YMRS-scores: not manic < 12, hypomanic < 21, manic < 30. Average IDS-C sum scores in manic or hypomanic states were significantly higher (p < .001) than for non-manic states. (Hypo)manic female patients were likely to show more depressive symptoms than males (p < .001). Similar results were obtained when only the core items of the YMRS or only the number of depressive symptoms were considered. Analyzing the frequency of (hypo)manic mixed states applying a proxy of the DSM-5 mixed features specifier extracted from the IDS-C, we found that almost 50% of the (hypo)manic group visits fulfilled DSM-5 mixed features specifier criteria.

**Conclusion:**

Subjects with a higher manic symptom load are also significantly more likely to experience a higher number of depressive symptoms. Mania and depression are not opposing poles of bipolarity but complement each other.

**Supplementary Information:**

The online version contains supplementary material available at 10.1186/s40345-021-00241-5.

## Introduction

Depressive symptomatology during a manic episode (also termed mixed, depressive, or dysphoric mania) is commonly seen in daily practice (Vieta and Valenti 2013) and, compared to pure mania, predicts a course of bipolar disorder (BD) with a younger age at onset, more frequent and longer episodes, more treatment resistance with delayed symptomatic remission, more frequent relapses, suicidality and suicidal acts, irritability, anxiety and substance abuse comorbidity (Grunze et al. 2018). According to the Systematic Treatment Enhancement Program for Bipolar Disorder (STEP-BD) study mixed states are less commonly experienced than euthymic and depressed states, but more temporally unstable, and uniquely associated with rapid cycling, substance use, and psychosis (Prisciandaro et al. 2019). In addition, quality of life, functioning in several domains, e.g., self-esteem, family, love and social life, physical wellbeing, and working capability seem to be severely affected by mixed states (Lee Mortensen et al. 2015).

Older studies applying research-based categorial definitions of mixed mania (Cincinnati criteria, Pisa criteria) indicate that relevant depressive symptoms, i.e. fulfilling the respective mixed features criteria, are present in about 30–40% of acutely manic patients (Akiskal et al. 2000; Hantouche et al. 2006; McElroy et al. 1992) whereas using full Diagnostic and Statistical manual, 4th edition (DSM-IV TR) criteria (American Psychiatric Association 1994) brings the prevalence down to 6.7% (Hantouche et al. 2006). Acknowledging that categorial DSM-IV criteria of mixed mania are too restrictive for deriving meaningful clinical implications, Diagnostic and Statistical manual, 5th edition (DSM-5) (American Psychiatric Association 2013) introduced a more dimensional approach defining depressive symptoms in manic patients and manic symptoms in depression as specifier, requiring ≥ 3 core symptoms of the opposite polarity. First results of studies using proxies for the DSM-5 mixed feature specifier suggest that again about 40% of acutely manic bipolar patients fulfil mixed features specifier criteria (McIntyre et al. 2013; Reinares et al. 2015; Vieta et al. 2014; Young and Eberhard 2015). Mixed mania across definitions appears to be more frequent in adolescents (Birmaher et al. 2006; Geller et al. 2004) and in patients with a high number of previous episodes (Gonzalez-Pinto et al. 2011; Mazzarini et al. 2018), especially in females (Kessing 2008).

The Stanley Foundation Bipolar Treatment Outcome Network (SFBN) recruited 935 bipolar outpatients from 1995 to 2002 from four sites in the United States and three in the Netherlands and Germany. Together with the “Systematic treatment enhancement program for bipolar disorder” (STEP-BD) (Sachs et al. 2003) the SFBN constitutes one of the largest, naturalistic and prospective follow-up studies in bipolar patients. The NFS was funded from 1995 until end of 2002. When funding was discontinued, the database was closed but the international group of investigators continued to analyse the wealth of data together as the Bipolar Collaborative Network (BCN). SFBN participants were rated extensively with several psychometric scales (Leverich et al. 2001), including daily self- and clinician ratings on the National Institute of Mental Health-Life Chart Method (LCM) (Born et al. 2014; Denicoff et al. 1997; Leverich and Post 1996). Having simultaneous ratings at defined time points enables us to examine the temporal relationship between diverse symptom domains, e.g., mania, depression, psychosis, functionality, and quality of life. Examples include previous analyses of depressive symptoms in hypomania (Suppes et al. 2005), manic symptoms while depressed (Miller et al. 2016) or the influence of subsyndromal symptoms on functionality (Altshuler et al. 2006).

## Aims of the study

This post-hoc analysis was conducted to clarify the relationship between manic and depressive symptom severity in acutely manic states. The term bipolar suggests that mania and depression do not co-occur. With the awareness of our previous study in hypomanic patients showing a reasonable depressive symptom load (Suppes et al. 2005), and after evaluating our so-called ODD sample (see “Methods” and “Results”) we post-hoc formulated and tested the following hypotheses:

1. Primary hypothesis: Patients in a manic state have higher Inventory of Depressive Symptoms—Clinician Version (IDS-C) values than patients in a non-manic state.
2. Secondary hypothesis: Patients in a hypomanic state have higher Inventory of Depressive Symptoms—Clinician Version (IDS-C) values than patients in a non-manic state.

## Methods

### Data collection

Subjects were drawn from an adult sample with bipolar disorder who participated in the SFBN after signing informed consent. Inclusion criteria for entry into the network and participating in the naturalistic follow-up study (NFS) were the diagnosis of either bipolar I, bipolar II, bipolar not otherwise specified or schizoaffective disorder, bipolar subtype according to the DSM-IV TR (American Psychiatric Association 1994), willingness to attend at least monthly visits and to participate in cross-sectional psychometric self- and clinician ratings and prospective life charting (Leverich et al. 2001). The only exclusion criteria were current active substance abuse or imminent suicidal threat. All diagnoses were based on the DSM-IV-TR criteria set and confirmed by the Structured Clinical Interview for DSM IV (First and Gibbon 2004).

**Fig. 1:**
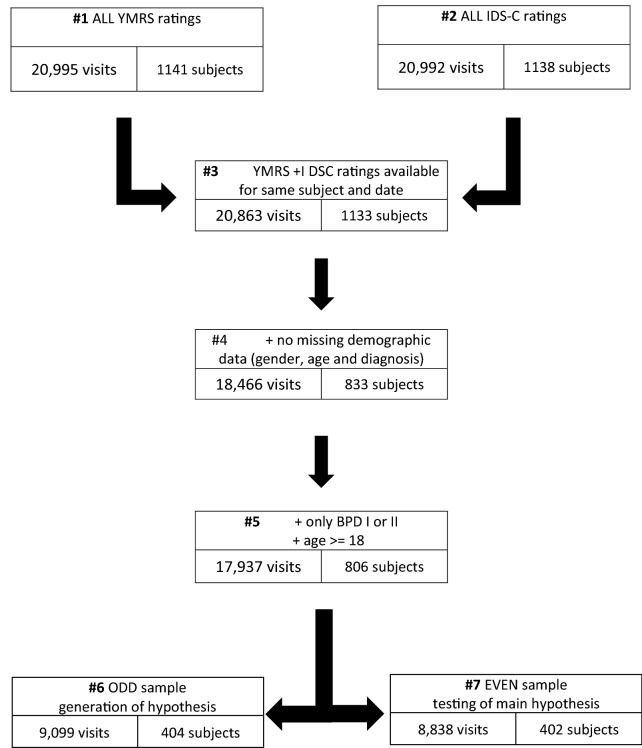
Flow chart of data selection

The detailed procedure of the NFS protocol has been reported elsewhere (Born et al. 2014; Leverich et al. 2001). IDS-C (Rush et al. 1986) and Young Mania Rating Scale (YMRS, (Young et al. 1978)) ratings were collected in real-time at each visit as part of the routine assessments. In addition to the NFS participants, this analysis also included subjects who volunteered only for specific intervention trials with YMRS and IDS-C measurements. Thus, this sample slightly differs from those in previous reports from our group on mixed features (Miller et al. 2016; Suppes et al. 2005). Figure 1 illustrates the sequential flow of selection.

### Data selection

This post-hoc analysis included only subjects with a diagnosis of bipolar I or II disorder. The final sample consisted of 806 subjects representing 17,937 visits with corresponding YMRS and IDS-C ratings collected at the same occasion. The average number of visits per year was 7.6 (SD ± 4.4), and the average duration of participation was 2.9 years (SD ± 1.8).

In the EVEN sample used for verification (see below) we a priori excluded those with a total YMRS rating of ≥ 30 (0.2%) after observing in the ODD sample a high variance with extreme ratings in this small group distorting regression models and raising considerable doubt how reliable depressive symptoms have been reported while in a highly excited manic state.

### Rating scales

The YMRS consists of 11 clinician-rated items measuring severity of distinct manic symptoms over the last 2 weeks. Some of the YMRS items overlap with symptoms observable in depression. To avoid overlap with depression rating scales, the so-called composite "YMRS core-score" consisting of the four items irritability, speech (rate and amount), content and disruptive-aggressive behavior has been created (Ketter et al. 2007) and also been used in subsequent acute mania studies, e.g., (Earley et al. 2018; Vieta et al. 2015). However, the 4 item core score is still not free of distortion as the item “irritability” is also included in the IDS-C. Therefore, we additionally tested a three item YMRS core score consisting of speech (rate and amount), content and disruptive-aggressive behavior only.

In this post-hoc analysis, both the full YMRS and the abbreviated core versions were applied. The four core items are rated with 0 to 8 points, the 7 other items with 0 to 4 points. Thus, the core items account for 32 of the maximum possible score of 60 points in the 4-item YMRS core score, and for 24 of 60 points in the 3-item YMRS core score.

Subjects were divided into three a priori defined groups according to manic severity. A YMRS total score of 12 to 20.5 points was counted as a hypomanic state, a sum score of ≥ 21 points as a manic state and a sum score of less than 12 points was considered as a non-manic state. Using only the YMRS core items for analysis, cut-offs between non-manic, hypomanic and manic were adapted accordingly (Table 1).

**Table 1:** Definition of manic, hypomanic and non-manic state by sum scores of all YMRS-items, 4 core YMRS-items and 3 core YMRS-items YMRS-items and core YMRS-items

State	YMRS-Score (total)	YMRS-Score (4 core-items)	YMRS-Score (3 core-items)
Non-manic	0–11.5	0–5.5	0–4
Hypomanic	12–20.5	6–10	4.5–7.5
Manic	21–29.5	10.5–14.5	8–11.5

The IDS-C consists of 30 items including the DSM-IV criteria for depression, but the scale also covers so called atypical symptoms of depression as weight gain, increased appetite and hypersomnia not rare in bipolar depression (Mitchell et al. 2008). Items are rated taking the last seven days into account. Each item is rated with 0 to 3 points (0, 1, 2 and 3 for no, mild, moderate or severe symptom load, respectively). A sum score of 16 is used as a cut-off value between depression and euthymia (Rush et al. 1996).

Across the four U.S. and the three European sites, interrater reliability was regularly assessed, and rater training was reinforced as needed to maintain consistent performance (kappa values were 0.7 for the YMRS and 0.85 for the IDS-C).

### Data analysis

A split sample approach was applied with subjects divided quasi-randomly into two independent groups using the registration number. Data from subjects with odd registration numbers (ODD-Sample) were used for evaluation/hypothesis building; data from subjects with even registration numbers (EVEN-Sample) constituted the verification/hypothesis testing sample. For preliminary evaluation and hypothesis building, various charting techniques were used to visualize the data from the ODD-Sample, including smoothing and normalization. To test hypotheses derived from ODD-Sample and verification we compared average IDS-C score ratings of non-manic, hypomanic and manic subjects of the EVEN-Sample. After verification of the primary and secondary hypotheses, additional analyses were conducted using the full data set (ODD and EVEN sample).

The database of the SFBN is located on an SQL-server (DB2 Universal Database version 8.2). Data were extracted using standard SQL functions (AVG, STDDEV, COUNT). All graphs were produced with Microsoft Excel 2003. T-tests and ANOVA were calculated with RStudio (Version 1.2.5033, RStudio Inc, Boston, Massachusetts). Considering that there might be a possible overlap of depressive and (hypo)manic symptoms as rated with the scales we tested for both—the total score and only the core items of the YMRS as mentioned above. Categorical variables were reported as frequencies and compared using the chi-square test, while continuous variables were reported as mean ± standard deviation (SD) and compared using the t test. The level of significance was set at p ≤ 0.05 for the primary and secondary hypotheses, where a compensation for multiple comparisons was not necessary, because of the type of dependency of the subgroups tested. For the additional analyses, following Bonferroni correction the level of significance was set at p ≤ 0.01 to compensate for multiple comparisons.

## Results

Complete data sets were available for 806 subjects. Mean age was 40 ± 12 years (range 18–81 years), and 460 subjects (57.1%) were female. Six hundred twenty-four were diagnosed with bipolar I disorder (77.4%) and 182 with bipolar II (22.6%). 17,937 visits of these subjects were available with corresponding YMRS and IDS-C, both rated at the same occasion. Female subjects contributed 9583 visits (53.4%) for analysis of the total score of the YMRS and 9593 visits (53.5%) for the analysis of the YMRS core items.

There were no significant demographic differences between the ODD and the EVEN sample. The ODD sample included 404 subjects with a mean age of 40 ± 12 years (range 18–81), and 235 subjects (58.2%) were female. In this sample, 320 subjects (79.2%) were diagnosed with bipolar I disorder. The EVEN sample included 402 subjects with a mean age of 40 ± 11 years (range 18–76), and 225 subjects (56.0%) were female. In this sample, 304 subjects (75.6%) were diagnosed with bipolar I disorder. 9099 (50.7%) visits were available from subjects with an odd registration number, 8838 (49.3%) visits were available from subjects with an even registration number.

Inspection of the plots “Average IDS-C score by YMRS score “and “Average IDS-C score by core-YMRS score “ suggested increasing severity of depressive symptoms with increasing YMRS rating scores in the ODDS sample (see Additional file 1: Fig. S5 and Fig. S6). Subsequently, we tested our primary and secondary hypothesis in the EVEN sample.

For verification of our hypotheses “(1) Subjects in a manic state have higher IDS-C-values than patients in a non-manic state” and “(2) Subjects in a hypomanic state have higher IDS-C values than patients in anon-manic state”, data were stratified into three groups defined by YMRS-scores (see Table 1). Average IDS-C Scores of these groups were compared with the t-Test.

### Using YMRS total score

When grouping by YMRS total score, 8,102 YMRS ratings (92.3%) felt into the non-manic range between 0 and 11.5 points, 583 YMRS ratings (6.6%) in the hypomanic range between 12 and 20.5 points and 93 YMRS ratings (1.1%) in the manic range between 21 and 29.5 points. 18 YMRS-scores (0.2%) with ≥ 30 points (total) were excluded from final analysis as we were in reasonable doubt how reliable depressive symptoms have been reported while in a highly excited manic state. Forty-two visits (0.5%) had to be excluded because of missing data.

In the non-manic group of the EVEN-Sample the mean IDS-C score was 15.75 ± 12.6 points (range 0–66), in the hypomanic group 19.06 ± 12.4 points (range 0–63) and in the manic group 18.29 ± 11.9 points (range 0–59) (Table 2).

**Table 2:** Mean IDS-C scores for the YMRS-groups (total and core items) EVEN SAMPLE

State	Average IDS-Score ± SD grouping by total	Average IDS-Score ± SD grouping by 4 core items	Average IDS-Score ± SD grouping by 3 core items
Non-manic	15.75 ± 12.59	15.46 ± 12.42	15.73 ± 12.52
Hypomanic	19.06 ± 12.43	20.76 ± 13.12	17.94 ± 12.45
Manic	18.29 ± 11.90	21.26 ± 13.23	21.73 ± 13.93

For the YMRS total score, the difference between (hypo)manic groups and the non-manic reached statistical significance (p < 0.001 for hypomanic vs non-manic, and p < 0.05 for manic vs non-manic) (Table 3).

**Table 3:** Differences in mean IDS-C scores for the YMRS-groups (total and core items) EVEN SAMPLE

State	Average IDS-Score ± SD Grouping by total YMRS	Average IDS-Score ± SD Grouping by YMRS 4 core items	Average IDS-Score ± SD Grouping by YMRS 3 core items
Hypomanic vs non-manic	19.06–15.75	20.76–15.46	17.94–15.73
Difference [95% CI]	3.3 [2.26; 4.36]	5.3 [4.32; 6.28]	2.21 [0.99; 3.44]
Significance (t-value)	*** (t = 6.2)	*** (t = − 10.6)	*** (t = 3.6)
Manic vs non-manic	18.29–15.75	15.46–12.42	15.73–12.52
Difference [95% CI]	2.54 [0.05–5.01]	*** (t = − 4.7)	*** (t = 6.4)
Significance (t-value)	* (t = 2.0)	21.26 ± 13.23	21.73 ± 13.93

*p < 0.05; ***p < 0.001; 95% CI = 95% confidence interval (two sided)

### Using YMRS core-score (4-item and 3-item)

Using the YMRS total score may confound results as there is some overlap with depressive symptom rating scales, including the IDS-C, (e.g., decreased sleep could be due to either depression or (hypo)mania). Using the sum score of the four YMRS core items might minimize this risk. However, as irritability is rated both in the 4-item YMRS core and the IDS-C, we also checked results for a 3-item YMRS core (see “Methods” section).

We identified 7914 4-item YMRS core scores (90.1%) in the non-manic range between 0 and 5.5 points, 748 4-item YMRS core scores (8.5%) in the hypomanic range between 6 and 10 points and 119 4-item YMRS core scores (1.4%) in the manic range between 10.5 and 14.5 points. 39 4-item YMRS core scores (0.4%) with ≥ 15 points (total) were excluded as we were in reasonable doubt how reliable depressive symptoms have been reported while in a highly excited manic state, 18 visits (0.2%) had to be excluded because of missing data.

In the 4-item YMRS core score defined non-manic group of the EVEN-Sample the mean IDS-C score was 15.46 ± 12.42 points (range 0–64), in the hypomanic group 20.76 ± 13.12 points (range 0–66) and in the manic group 21,26 ± 13.23 points (range 0–59) (Table 2). All differences between all groups reached significance (p < 0.001) (Table 3).

Using only the sum score of the other 3 YMRS core items, we identified 8129 3-item YMRS core scores (92.17%) in the non-manic range between 0 and 4 points, 420 3-item YMRS core scores (4.76%) in the hypomanic range between 4.5 and 7.5 points and 230 3-item YMRS core scores (2.6%) in the manic range between 8 and 11.5 points. 39 3-item YMRS core scores (0.46%) with ≥ 12 points (total) were excluded as we were in reasonable doubt how reliable depressive symptoms have been reported while in a highly excited manic state, 18 visits (0.2%) had to be excluded because of missing data.

In the 3-item YMRS core score defined non-manic group of the EVEN-Sample the mean IDS-C score was 15.73 ± 12.52 points (range 0–62), in the hypomanic group 17.94 ± 12. points (range 0–64) and in the manic group 21.73 ± 13.93 points (range 0–64) (Table 2). All differences between all groups reached significance (p < 0.001) (Table 3).

Summarizing these results, mean IDS-C scores related to the severity of the manic state as measured with the core items of the YMRS (Table 2). When using the total YMRS score instead of the YMRS core score, we observed significantly higher depressive symptom severity in hypomanic and manic subjects compared to non-manic subjects, too, but no significant difference between hypomanic and manic probands.

### Additional analyses in the full sample

After verification of the primary and secondary hypotheses, additional analyses were conducted using the full data set (ODD and EVEN sample).

#### IDS-C score in relation to mood state (euthymic, hypomanic, manic)

**Fig. 2:**
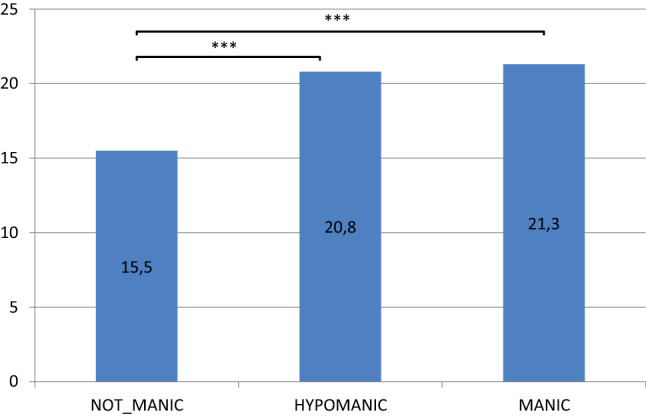
Average IDS-C scores by state (based on 4-item YMRS core-score), EVEN sample

Plotting the average IDS-C sum score for the full data set as a function of the categories non-manic, hypomanic and manic as defined by the 4-item YMRS core score demonstrates significant differences between groups (p < 0.001) with the manic group scoring highest, and the non-manic group lowest on the IDS-C (Fig. 2).

#### Number of depressive symptoms

**Fig. 3:**
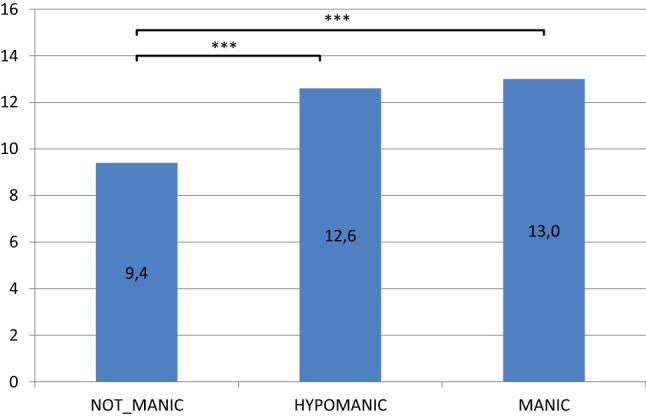
Average IDS-C-Symptom count by state (based on 4-item YMRS core-score), EVEN sample. ***p < 0.001

Plotting only the IDS-C sum score as a function of the YMRS score supplies an estimate of severity of depression, but does not reflect the full symptomatology, e.g., how many mental, social and physical domains are affected. Results for the mean number of depressive symptoms (IDS-C items scoring ≥ 1) in the non-manic, hypomanic and manic group as defined by 4-item YMRS core-scores are shown in Fig. 3. Table 4 illustrates that these results are stable and significant for all three definitions of mood state, by total YMRS score, 4-item and 3-item YMRS core score.

**Table 4:** Mean IDS-C SYMPTOMCOUNT for the YMRS-groups (total and core items) in the full sample

State	Average IDS-Symptom Count ± SD Grouping by total	Average IDS-Symptom Count ± SD Grouping by 4 core items	Average IDS-Symptom Count ± SD Grouping by 3 core items
Non-manic vs hypomanic	9.5 ± 6.5 *** (t = 12.1) 11.9 ± 6.1	9.4 ± 6.5 *** (t = 18.4) 12.6 ± 6.2	9.5 ± 6.5 *** (t = 6.8) 11.1 ± 6.3
Non-manic vs manic	9.5 ± 6.5 ** (t = 3.0) 10.9 ± 6.1	9.4 ± 6.5 *** (t = 9.0) 13.0 ± 6.3	9.4 ± 6.5 *** (t = 9.4) 12.5 ± 6.2

***p < 0.001, **p < 0.01

#### Gender effect on number of depressive symptoms

**Fig. 4:**
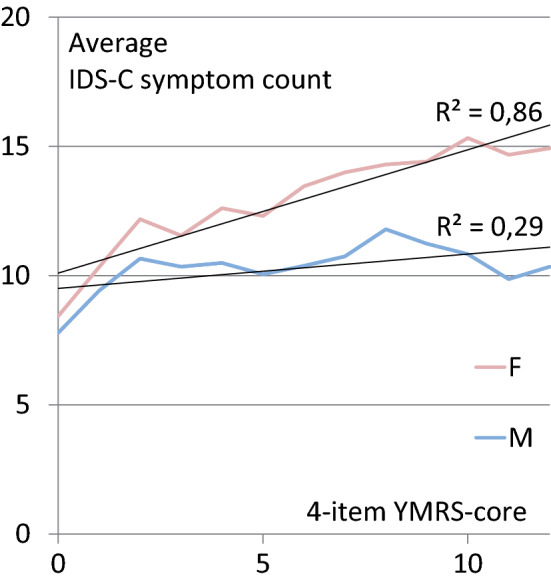
Average IDS-C-Score by 4 item YMRS CORE SCORE (male vs female)

Previous analyses showed a female preponderance for mixed hypomania (Suppes et al. 2005) and numerically also for mixed mania (Vieta et al. 2014). To test whether this is also true in our study we compared numbers of IDS-C items scoring ≥ 1 as a function of manic severity separately for female and male subjects. Female patients were affected by more depressive symptoms than males, especially when manic symptoms were more severe. We observed a linear increase of depressive symptomatology with severity of manic core symptoms (average IDS-C-symptom count vs 4-item YMRS core score, R2 = 0.86), but not in males (R2 = 0.29) (Fig. 4). Thus, the increase of the number of depressive symptoms associated with an increase of YMRS-core scores was mainly carried by women. Factorial analysis of variance testing showed highly significant (p < 0.001) effects for all 3 factors, that is (1) YMRS 4 item core score (2) gender as well as (3) the combination of YMRS 4 item core score and gender.

#### Number of occasions and subjects fulfilling DSM-5 hypomanic or manic with mixed features specifier criteria

Finally, we examined at how many occasions (visits) DSM-5 hypomanic or manic with mixed features specifier criteria were likely to be satisfied, and the corresponding number of subjects. As a proxy we assigned the six depressive A-criteria listed in DSM-5 to corresponding specific IDS-C items (Table 5). Symptom criterion was counted as satisfied with a score of at least 1, except criterion 1 where the corresponding IDS-C items 5 or 10 should score at least 2 to satisfy also the duration criterion of DSM-5 (“majority of days”). To criterion 1, we also assigned two IDS-C items as sadness alone (item 5) might not cover all flavors of depressed mood; however, for fulfilling DSM-5 criterion 1 it was sufficient to score at least 2 in either item 5 or 10.

**Table 5:** Assignment of the six DSM-5 depressive symptoms for (hypo)mania with mixed features specifier to selected IDS-C items

DSM-5 depressive symptom (abbreviated)	Corresponding IDS-C item
1. Prominent dysphoria or depressed mood	5 (sad mood) * or 10*
2. Diminished interest or pleasure an all, or almost all, activities	19 (involvement) or 21 (pleasure/enjoyment)
3. Psychomotor retardation nearly every day	23 (psychomotor slowing)
4. Fatigue or loss of energy	20 ((energy/fatiguability)
5. Feelings of worthlessness or excessive or inappropriate guilt	16 (outlook-self)
6. Recurrent thoughts of death	18 (suicidal ideation)

*A minimum score of 2 is needed to satisfy criterion

Using the YMRS total scores for assignment to the non-manic, hypomanic and manic group, we found that 432 visits in the hypomanic group (40,6%) and 58 visits in the manic group (32,2%) fulfilled DSM-5 criteria for a (hypo)manic episode with mixed features specifier (≥ 3 depressive symptoms).

Applying the 4-item-YMRS core score to group subjects, we found that 652 visits in the hypomanic group (47.7%) and 121 visits in the manic group (48%) fulfilled DSM-5 criteria for a (hypo)manic episode with mixed features specifier (≥ 3 depressive symptoms). There was no significant difference at p ≤ 0.01 between the manic and hypomanic group. Examining gender preponderance, we found that in the hypomanic group 55.2% of visits of females and 39.1% of visits of males were likely to satisfy mixed specifier criteria, and in the manic group 53.5% of visits of females and 40.7% of visits of males. Differences between visits of females and males were significant at p ≤ 0.001 in the hypomanic group, but with the relatively low number of visits only a trend (p = 0.45) was observed in the manic group.

Next, we examined how many (hypo)manic subjects fulfilled the mixed feature specifier at least at one visit. As a result, we found that 272 subjects in the hypomanic group (64%) and 77 subjects in the manic group (50%) as defined by 4-item YMRS core score fulfilled DSM-5 criteria for a (hypo)manic episode with mixed features specifier (≥ 3 depressive symptoms) at some point in time. There was a significant difference at p ≤ 0.01 between the hypomanic and manic group. Examining gender preponderance, we found that in the hypomanic group 66.7% of females and 60.8% of males were likely to satisfy mixed specifier criteria at some point in time, and in the manic group 51.1% of females and 48.5% of males. There was no significant difference between female and male subjects.

#### Non-mood symptoms predict more severe depression in (hypo)manic patients

Previous work suggests that the non-mood-symptoms anxiety, agitation and irritability are more prevalent in manic patients suffering from a higher depressive burden and fulfilling DSM-5 mixed feature specifier (Suppes et al. 2017; Vieta et al. 2014). In our sample, we examined the correlation between severity of agitation and irritability vs. average IDS-C score. As a proxy for the degree of agitation, we used the YMRS item 2 and defined a score 0–2 as “no or low degree of agitation”, and a score > 2 as “agitated”. As a proxy for the degree of irritability, we used the YMRS item 5 and defined a score 0–4 as “no or low degree of irritability”, and a score > 4 as “irritable”. We could not test for anxiety as there is no appropriate YMRS item as an equivalent. Finally, we compared the average IDS-C value in the group of “High scorer for agitation and irritability”, scoring > 2 points for item 2 and > 4 points for YMRS item 5, against the rest of the sample. Confirming previous studies, we found that scoring high on YMRS item 2 (Agitation) or/and 5 (Irritability) significantly predicted more severe depression in (hypo)manic subjects (Table 6).

**Table 6:** Scoring high on YMRS item 2 (Agitation) or/and 5 (Irritability) predicted more severe depression in (hypo)manic subjects

YMRS item	IDS-c score for no or low degree of agitation or irritability	IDS-c score for agitated or irritable	p-Value
Agitation (item 2)	20.78	22.38	0.04
Irritability (item 5)	20.47	31.96	< 0.001

	IDS-c score for no or low degree of both agitation and irritability	IDS-c score for agitated or irritable	p-Value
Agitation and irritability	20.07	24.12	< 0.001

	IDS-c score for no or low degree of both agitation and irritability	IDS-c score for both agitated and irritable	p-Value
Agitation and irritability	20.07	30.05	< 0.003

#### Panic and agitation increase with hypomania

For clinicians it is relevant to know which depressive symptoms have the strongest association with the DSM-5 mixed feature specifier in (hypo-)mania. Therefore, we compared the average values for all IDS-C items between the 3 groups non-manic, hypomanic and manic (see Table 1) for subjects fulfilling criteria for the depressive mixed features specifier.

Compared to the non-manic group, the hypomanic group showed more than 50% increase of the average item score for the IDS-C items interpersonal sensitivity (+ 54%), early morning insomnia (+ 56%), irritable mood (+ 78%), panic/phobic Symptoms (+ 100%), psychomotor agitation (+ 120%).

Compared to the non-manic group, the manic group showed a greater than 50% increase of the average item score for the IDS-C items for anxious mood (+ 50%), sleep onset insomnia (+ 51%), sympathetic arousal (+ 57%), interpersonal sensitivity (+ 66%), suicidal ideation (+ 67%), mid-nocturnal insomnia (+ 67%), weight decrease (+ 67%), panic/phobic symptoms (+ 107%), irritable Mood (+ 114%), early morning insomnia (+ 128%), and psychomotor agitation (+ 206%).

Compared to the hypomanic group, the manic group showed the biggest increase of the average item score for the IDS-C items for mid-nocturnal Insomnia (+ 27%), suicidal ideation (+ 30%), weight decrease (+ 34%), psychomotor agitation (+ 39%) and early morning insomnia (+ 46%),

## Discussion

This analysis of a large bipolar I and II cohort participating in the SFBN demonstrates a high depressive burden in subjects fulfilling symptomatic criteria for mania or hypomania as measured with the YMRS total and two different YMRS core sets (with four and with three items). Summarising the different studies looking into gender preference, mixed manic episodes across definitions (mixed mania, dysphoric mania or the manic-depressive dimension) appears more common in females than in males, with the ratio being about 60: 40 (Gonzalez-Pinto et al., 2011), and was also observed in this post-hoc analysis. In the study by Suppes et al. (2005) severity of depressive symptoms was not significantly greater as a function of more severe hypomanic or manic symptoms using only YMRS total scores, but rather resembled a bell-shaped curve. We also observed a flattening of the curve with higher YMRS total scores, with no significant difference in IDS-C scores between hypomanic and manic patients. However, when applying the 4 item YMRS core score we observed a linear increase of depressive severity in females, but not in males, with hypomanic and manic severity. Here we observed not only a significant difference in depressive symptom severity between non-manic and hypomanic or manic patients, but also between hypomanic and manic subjects with a concomitant increase of depressive burden with the severity of mania ratings. The YMRS core score has been frequently used in other studies, however, when used in conjunction with a depression rating such as the IDS-C, the item “irritability” overlaps between scales and has greater weight in the YMRS core set compared to the YMRS due to the smaller number of items. This double counting may, to some degree, skew results, more when using the YMRS core than the full YMRS. However, when applying a 3-item YMRS core leaving out irritability, results remained similar and statistically significant.

Analysing the frequency of manic mixed states applying a proxy of the DSM-5 mixed features specifier extracted from the IDS-C, we found that almost 50% of visits fulfilled criteria and 50–64% of patients at some point in time. The approximate equality of numbers suggests that not a few patients with highly frequent mixed states drive the number of “mixed” visits, but that mixed features do have a wide distribution within our cohort. One of the concerns regarding the validity of the total YMRS score is that depressive symptoms might contribute to and confound the total YMRS score. Different from the total YMRS score, the 4-item YMRS core score excludes all depressive symptoms. If depressive symptoms contribute to the total YMRS score, but do not contribute to the 4-item YMRS score, a lower rate of depressive symptoms associated with manic 4-item YMRS scores appears plausible. On the other hand, if the 4-item YMRS core score separates manic states from non-manic states better than the total YMRS score, and if manic states are associated with more mixed features that non-manic states, it is also plausible to expect a higher rate of mixed features using the 4-item core score opposed to the total YMRS score. Our results, indicating a higher rate of mixed features using the 4-item YMRS core score, would favour the hypothesis that the 4-item YMRS core score is more sensitive than the total YMRS score in separating manic states from non-manic states, and that manic states are associated with more depressive features than non-manic states.

In the IMPACT study (Vieta et al. 2014) non-mood-symptoms as anxiety, agitation and irritability that are known to correlate with inferior outcomes were also more prevalent in the mixed mania group. Thus, the presence of anxiety with irritability or agitation might be an indicator for inferior outcomes in manic patients with mixed features specifier (Vieta et al., 2014), despite the fact that these symptoms are not part of the definition of a mixed feature specifier. Our post-hoc analysis supported that scoring high on YMRS item 2 (Agitation) or/and 5 (Irritability) significantly predicted more severe depression in (hypo)manic subjects. In line with this, a post-hoc analysis of a Phase III medication study also found that the presence of anxiety, irritability and agitation in bipolar mania might enable physicians at the same time to identify patients with more severe depressive symptoms (Suppes et al. 2017). But again, the results for YMRS item 5 (irritability) as a predictor for higher depressive burden needs to interpreted with caution as irritable manic patients also score automatically on IDS-C item 6 (irritable mood).

The abundance of depressive symptoms in hypomanic subjects confirms our previous findings that mixed hypomania is common in patients with symptoms of hypomania and particularly common in women (Suppes et al. 2005), even when only manic core items are considered and overlapping items with depression are omitted. In a previous report on the SFBN cohort we also found that mixed depression, defined as an IDS-C score ≥ 15 and a YMRS score > 2 and < 12 at the same visit, was observed in 43.5% of visits when subjects were depressed (Miller et al. 2016). These three studies together demonstrate again clearly that mania and depression are not opposing poles of bipolarity but complement each other.

Different from simplified neurobiological concepts, psychodynamic and psychoanalytic concepts do not see a contradiction in a coexistence of depression and mania in what they consider “stable” mixed states (Maggini et al. 2000). They consider depression as the manifestation of losses (e.g., the loss of self-esteem and the sense of worthlessness) and mania serves as a defence against the feelings of depression (Klein 1960). Instability of mixed states, manifesting itself, e.g., as ultradian rapid cycling, might be linked to temperament intruding into an episode of opposite polarity (Akiskal 1992; Marneros 2001).

Limitations of our study include the post-hoc nature of the analysis. Data were collected prospectively without any a priori intention of this analysis for mixed features and we a priori decided for a split sample approach, to make a clear separation between evaluator analysis / hypothesis generation on the one side and hypothesis testing / evidence generation on the other side, but the primary and secondary hypotheses that were tested were generated while aware of a previous, similar evaluation in an overlapping population (Suppes et al. 2005). To minimize overlap between YMRS and IDS-C, we created a 3-item YMRS core set that needs further evaluation of its validity. As far as our results about the proportion of patients and visits fulfilling DSM 5 specifier are concerned, we used a self-developed assignment of specific IDS-C items to mixed feature specifier criteria as a proxy that also has not yet been prospectively evaluated for its validity. Additional analyses suffer from the problems of repeated testing and are reported as findings, in contrast to the evidence generated by testing the primary and secondary hypotheses. A full analysis of the interdependency of manic and depressive symptoms is beyond the scope of this paper. The description of percentual change of average IDS-C values between non-manic, hypomanic and manic state based on this mixed feature specifier is descriptive only, no probabilities have been calculated.

## Conclusion

Subjects with a higher manic symptom load were also significantly more likely to experience a higher number of depressive symptoms. In women, we observed a linear increase of depressive burden with increasing YMRS core ratings, and females in general were significantly more likely to experience a larger number of depressive symptoms than males. Approximately half of the patients in the hypomanic and manic group were also likely to fulfill the DSM-5 mixed feature specifier, again with a significant female preponderance. The presence of agitation and irritability, individually and together, was a strong predictor for a higher depressive burden in hypomanic or manic subjects. Future research should consider mania and depression not as opposing poles of bipolarity but as complementing states of manic-depressive disorders.

## Supplementary Information

**Additional file 1: Figure S5.** Average IDS-C Score by YMRS score (ODD Sample). **Figure S6.** Average IDS-C Score by 4-item YMRS core score (ODD Sample).

## Data Availability

The datasets used and/or analysed during the current study are available from the senior author on reasonable request.

## Abbreviations

BCN: Bipolar Collaborative Network
BD: Bipolar disorder
DSM: Diagnostic and statistical manual
IDS-C: Inventory of Depressive Symptoms—Clinician Version
LCM: Life Chart Method
NFS: Naturalistic follow-up study
SFBN: Stanley Foundation Bipolar Treatment Outcome Network
Step-BD: Systematic Treatment Enhancement Program for Bipolar Disorder
YMRS: Young Mania Rating Scale
