# Supplementary material for: Mania and bipolar depression: complementing not opposing poles—a post-hoc analysis of mixed features in manic and hypomanic episodes
Source: Int J Bipolar Disord. 2021 Nov 16;9:36. doi: 10.1186/s40345-021-00241-5 (PMC8593087; doi:10.1186/s40345-021-00241-5)

Additional file 1

Figure S5: Average IDS-C Score by YMRS score (ODD Sample)

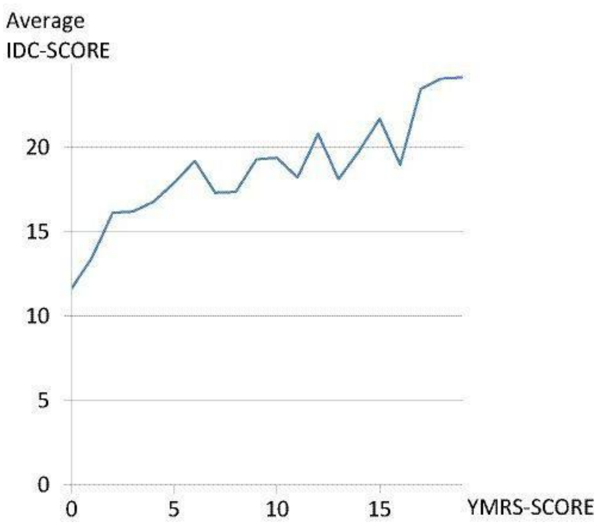

Figure S6: Average IDS-C Score by 4-item YMRS core score (ODD Sample)

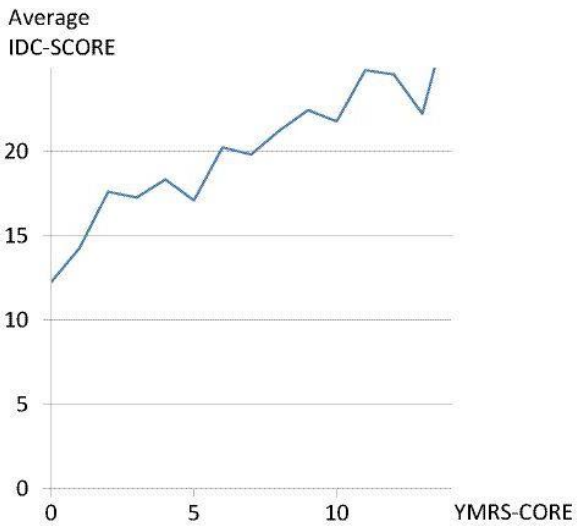

Supplement: Supplementary file 1 — Additional file 1: Figure S5. Average IDS-C Score by YMRS score (ODD Sample). Figure S6. Average IDS-C Score by 4-item YMRS core score (ODD Sample). [file 40345_2021_241_MOESM1_ESM.pdf]
